# The bile duct and liver cancer: ON-treatment surveillance of tumor evolution and response to systemic treatment (BILLIONSTARS) study

**DOI:** 10.1186/s12885-025-14429-w

**Published:** 2025-06-11

**Authors:** Philip Falk, Sofie Olsson Hau, Hedda Jacobsen, Jakob Eberhard, Caroline Williamsson, Karin Jirström

**Affiliations:** 1 Division of Oncology and Therapeutic Pathology, Department of Clinical Sciences Lund, Lund University, Lund, SE-221 85 Sweden; 2 Department of Hematology, Oncology and Radiation Physics, Skåne University Hospital, Lund, Sweden; 3 Department of Palliative Care and Advanced Home Health Care, Primary Health Care Skåne, Malmö, Sweden; 4 Division of Surgery, Department of Clinical Sciences Lund, Lund University, Lund, Sweden; 5 Department of Surgery, Skåne University Hospital, Lund, Sweden; 6 Department of Clinical Genetics, Pathology and Molecular Diagnostics, Skåne University Hospital, Lund, Sweden

**Keywords:** Hepatocellular carcinoma, Cholangiocellular carcinoma, Cholangiocarcinoma, Targeted therapy, Tumor heterogeneity, Tumor evolution

## Abstract

**Background:**

Malignancies of the liver and bile ducts are associated with high recurrence rates after surgery and poor prognosis when disseminated. Medical treatment has been improved in recent years, with chemotherapy, targeted therapy and checkpoint inhibitors offering opportunities to influence the course of the diseases. Many patients do not benefit from treatment, however, and predictive and prognostic markers are lacking. The BILe duct and LIver cancer: ON-treatment Surveillance of Tumor evolution And Response to Systemic treatment (BILLIONSTARS) study aims to map how molecular tumor characteristics and pathways of genetic evolution align with treatment response trajectories.

**Methods:**

The BILLIONSTARS study is a prospective, single arm observational study. Patients at Skåne University Hospital in Malmö/Lund and Central Hospital in Kristianstad diagnosed with hepatocellular carcinoma (HCC) or cholangiocarcinoma (CCC) who are to be recommended locoregional intervention by surgery, ablation, transarterial chemoembolization or selective internal radiation therapy and/or antitumoral medical treatment will be included. Tissue obtained in the clinical setting through surgery or biopsy along with tissue from research autopsies will be evaluated with targeted deep sequencing. Circulating tumor DNA (ctDNA) and other relevant biomarkers will be analyzed in blood samples obtained at different timepoints, depending on the type of treatment; before surgery or the start of systemic treatment, prior to each course of systemic treatment, if applicable, and at end of treatment.

**Discussion:**

The treatment field for HCC and CCC is evolving, thus improving outcomes for patients in the palliative setting. The efficacy of targeted therapy and checkpoint inhibition is however highly variable, and no predictive biomarkers have yet been established. The clinical course of the diseases is also highly variable and unpredictable. With this study, we hope to gain an increased insight into the various biological characteristics of these tumors, including novel potential treatment targets, as well as their spatial and temporal heterogeneity. By conducting research autopsies with comprehensive post-mortem sampling, we expect to further expand our understanding of the molecular events leading to terminal disease. The ultimate goal is to design better individualized and adaptive treatment strategies, thereby improving the outlook for patients with HCC or CCC.

**Trial registration:**

This study has been registered in clinicaltrials.gov as NCT06877637 Protocol version 1 March 2025

## Background

Hepatocellular carcinoma (HCC) and cholangiocarcinoma (CCC) are distinct, yet partially overlapping, diseases. Both come with a dismal prognosis for the majority of affected patients, although the advent of targeted therapies and checkpoint inhibitors have improved the chance of prolonged survival for some.

### Hepatocellular carcinoma

HCC comprises 75–85% of primary cancers in the liver [1] and the incidence is rising. Primary risk factors are chronic infection with hepatitis B or C virus, metabolic dysfunction-associated steatotic liver disease (MASLD) with or without diabetes mellitus type 2 and alcoholic liver disease [2]. The past decades have seen considerable epidemiological and geographical changes due to the advent of powerful antiviral therapies and lifestyle changes, but an increase in obesity and MASLD. The yearly incidence in Sweden is approximately 500 cases, and is increasing, particularly among men aged 50–65 years [3]Curative treatment includes resection, ablation or transplantation, with resection being the primary choice in patients with non-cirrhotic livers. In patients with smaller tumors (< 3 cm), resection and ablation appear to render similar survival rates, while ablation is associated with a shorter tumor free survival [4]. Even with seemingly radical resection, recurrence rates are high. Adjuvant or neoadjuvant systemic therapy does not yet have a clear place in HCC. Trials are ongoing in these settings. In disease beyond curative treatment but still liver confined, transarterial chemoembolization (TACE) or selective internal radiation therapy (SIRT) can be considered. In advanced disease, prolonged survival may be achieved with palliative systemic treatment with atezolizumab and bevacizumab in combination or tyrosine kinase inhibitors (TKI: s; sorafenib/lenvatinib), the former being superior both in terms of response rate, progression free survival (PFS) and overall survival (OS) compared to sorafenib [5]. Durvalumab and tremelimumab in combination has also been shown to be superior to sorafenib regarding response rate and OS, but not PFS [6], and ipilimumab in combination with nivolumab was superior to lenvatinib/sorafenib, with prolonged OS, in the Checkmate-9DW study [7]. These three regimens have not yet been compared head-to-head. The overall response rates with the three combination regimens were 30%, 20% and 36% respectively (atezo-bev, durva-trem, ipi-nivo) meaning that the majority of patients did not benefit from these treatments.

The pathogenicity of HCC differs between the various etiologies and give rise to different mutations and genomic alterations [8]. Viral hepatitis induced HCC tend to be more susceptible to treatment with TKI: s and PD-L1/VEGFR antibodies, but the precise biological mechanism for this has not been established [5, 9]. There is currently no established biomarker with the purpose of predicting response to immunotherapy or TKI: s in HCC.

### Cholangiocarcinoma

CCC encompasses a heterogenous constellation of tumors that can arise anywhere in the biliary tree. CCCs are divided into subgroups based on their anatomical origin; intrahepatic (iCCC), perihilar (pCCC), distal (dCCC) and gall bladder (GBC). The molecular characteristics differ significantly depending on the anatomic location. The yearly incidence in Sweden is approximately 500 cases, with a slight predominance for women, in particular regarding gall bladder cancer. Several risk factors have been linked to CCC; choledochal cysts, bile duct and gall stones, primary sclerosing cholangitis (PSC), cirrhosis, liver fluke, chronic hepatitis B or C [10–12]. Some risk factors apply to all subtypes whereas other are more specific to one subtype and more relevant in certain geographical areas. Many risk factors share the common feature of being linked to chronic inflammation in the biliary epithelium and bile stasis [13].

Neoadjuvant chemotherapy does not yet have a place in CCC except for within clinical trials. Published data indicate a benefit in particular for patients with risk factors, but there is still a great need for prospective trials [14]. As of yet, platinum combinations have not been shown to be superior to 5-FU regimens in the adjuvant setting [15]. In the palliative setting, cisplatin combined with gemcitabine appears to be superior to other regimens in terms of both OS and PFS [16], with oxaliplatin being a viable option to cisplatin [17]. The addition of PD1/PDL1-inhibitors (pembrolizumab/durvalumab) to gemcitabine plus cisplatin further enhance treatment efficacy, though modestly [18, 19].

Isocitrate dehydrogenase 1/2 (IDH1/2) mutations and Fibroblast growth receptor 2 (FGFR2) alterations are common in intrahepatic and perihilar CCC, and targetable with specific drugs, albeit with moderate effect [20, 21]. In contrast to other solid cancers, there are however no established markers predicting response to immunotherapy, except for dMMR/MSI-H tumors [22]. In certain countries, tumor mutational burden (TMB) ≥ 10 mut/Mb is approved as a selector for immunotherapy [23, 24], although such a threshold has not been shown to be appropriate for biliary tract tumors specifically.

Less common targetable genomic mutations and alterations occur in cholangiocarcinoma, with phase II studies with small sample sizes suggesting clinical efficacy, including BRAF V600E mutations [25], HER2 amplification [26–28] and TRK fusion [29].

### Mixed tumors

Mixed HCC-CCC are considered a separate entity. They are rare, arise in the liver, and have a particularly aggressive biology [30, 31]. There is a morphological spectrum between HCC and intrahepatic CCC, reflecting a common progenitor cell in a proportion of cases. Mixed tumors are classified as intrahepatic CCCs according to TNM8.

### Objectives

The aim of the BILe duct and LIver cancer: ON-treatment Surveillance of Tumor evolution And Response to Systemic treatment (BILLIONSTARS) study is to gain a deeper understanding of the molecular biology of HCC and CCC, and to delineate clinically relevant subgroups and novel diagnostic approaches to improve personalized treatment, and, ultimately, patient outcome. In parallel, we will study how the diseases and their medical interventions affect patients’ quality of life, to improve personalized care.

## Methods/design

### Study design and eligibility criteria

The BILLIONSTARS study is a prospective, single-arm observational study. Patients with clinically diagnosed HCC, mixed HCC/CCC, iCCC, pCCC, dCCC or gall bladder carcinoma planned to undergo surgery, ablation, transarterial chemoembolization or systemic treatment at Skåne University Hospital and Central Hospital in Kristianstad will be invited to participate. For patients planned to receive palliative systemic treatment, histological or cytological confirmation is required, except for patients with LI-RADS 5 lesions. Main exclusion criteria are age < 18 years, severe comorbidities or inability to comprehend study information. A total enrolment of 150 patients is planned. Enrolment will start in May 2025 and will be concluded by the end of 2029. The study has been registered in clinicaltrials.gov (NCT06877637).

Before start of treatment, the patients will receive information about the study by a physician and give their written informed consent. Tumor tissue will be obtained from surgical specimens, predominantly from formalin-fixed paraffin embedded (FFPE) tissue, but in some cases, fresh tissue will be obtained at surgery for cryopreservation to enable future preclinical studies. All blood samples will be taken by a dedicated research nurse in conjunction with the treatment visits. Plasma, serum and buffy coat will be isolated from blood samples, following standard fractionation, to enable analyses of circulating tumor DNA (ctDNA), and other biomarkers. Participants will be asked to fill in quality of life questionnaires (EORTC QLQ-C30 and EORTC-QLQ-HCC18/BIL21) at study start, and after three and six months, respectively.

Radiological and clinical follow-ups will be conducted according to clinical protocols or when deemed necessary by the clinician. Primary endpoint is OS, secondary endpoints are progression free survival and change in quality of life (EORTC QLQ-C30 + EORTC-QLQ-HCC18/BIL21).

The study will also include research autopsies. Patients are informed and provide their consent for inclusion in this part of the study when they are in a late palliative phase.

### Blood sampling

**Fig. 1:**
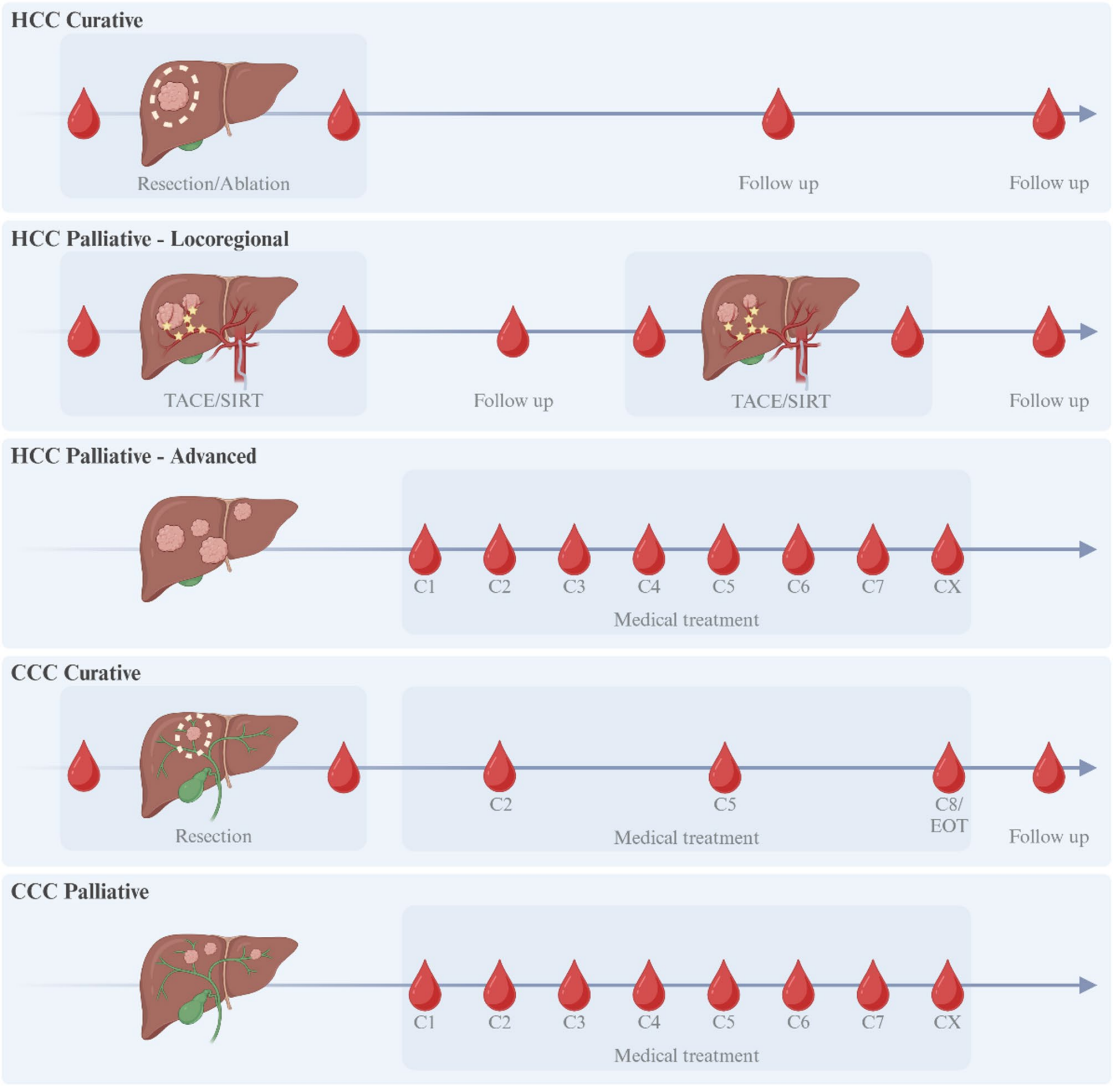
Time points for blood sampling. The blood droplets represent timing for blood sample collection for the different scenarios. Image created with https://www.biorender.com/

A graphical flowchart of the study is shown in Fig. 1, and an overview of the timepoints for blood sampling, depending on the tumor type and treatment intention, is provided in Tables 1, 2, 3, 4 and 5. For CCC patients planned for surgery, the pre-intervention sample will be taken prior to surgery and before start of chemotherapy. The ensuing samples will be collected before Capecitabin cycle 2, 5 and 8 and at follow-up, usually one year after surgery. For the HCC patients who generally do not receive adjuvant treatment, the blood samples will be collected before and after surgery, and with clinical follow-up 6 and 12 months after surgery. For the HCC patients receiving TACE, blood samples will be collected before the procedure and at clinical follow-up, initially after 4–6 weeks, later every three months for as long as additional TACE is considered, up to 12 months after the last procedure. For all patients receiving palliative systemic therapy, samples will be collected before start of treatment in conjunction with visits for the medical treatment, with each cycle. The end of treatment sample will be taken at the follow-up visit on the oncology department, with cessation of first line treatment or 1 year after treatment start, at the latest.

**Table 1:** Curative CCC

Investigation	Preop	Postop/prechem	C2	C5	C8/EOT	FU 1 y	
CT-scan	x				x	x	
Tumor specimen	x	x					
Physical examination	x	x	x	x	x	x	
Routine blood tests	x	x	x	x	x	x	
CA 19 − 9	x	x			x	x	
Blood samples investigation	x	x	x	x	x	x	
Quality of life survey		x		x	x	x	
Body weight	x	x	x	x	x	x	
Performance status	x	x	x	x	x	x	

Time points for acquisition of data from patients with CCC undergoing curative treatment. Inclusion and initial assessment are carried out at the surgical department. The follow-up visit after surgery and visit before oncological treatment (if applicable) lies in conjunction, and is the base for the second assessment. The ensuing time points for collection of data is before cycle 2 and 5 of Capecitabine and in conjunction with cessation of treatment after 6 months. A final assessment is being carried out 1 year after surgery, at the surgical department

**Table 2:** Palliative CCC (Gemcis)

Investigation	Prechem	C2-17	Evalu 3 mo	Evalu 6 mo	Evalu 9 mo	Evalu 12 mo
CT-scan	x		x	x	x	x
Tumor specimen	x					
Physical examination	x		x	x	x	x
Routine blood tests	x	x				
CA 19 − 9	x		x	x	x	x
Blood samples investigation	x	x				
Quality of life survey	x		x	x	x	x
Body weight	x	x	x	x	x	x
Performance status	x	x	x	x	x	x

Time points for acquisition of data from patients with CCC undergoing palliative treatment. Inclusion and baseline assessment is carried out at the oncology department before start of treatment. Before each treatment cycle routine blood tests and additional investigational blood samples are gathered. CT-scans and clinical follow-up occur usually every three months during treatment. Last follow-up is after 12 months, or with cessation of treatment

**Table 3:** HCC curative

Investigation	Preop	Post op	FU 1	FU 2			
CT-scan	x		x	x			
Tumor specimen		x					
Physical examination	x	x	x	x			
Routine blood tests	x	x	x	x			
CA 19 − 9, AFP	x	x	x	x			
Blood samples investigation	x	x	x	x			
Quality of life survey		x	x	x			
Body weight	x	x	x	x			
Performance status	x	x	x	x			

Time points for acquisition of data from patients with HCC undergoing curative treatment. Inclusion and initial assessment are carried out at the surgical department, with repeated assessment after surgery and at follow-up, usually six and twelve months after surgery

**Table 4:** HCC palliative - locoregional

Investigation	Pre proc 1	FU 1	Proc 2-x	FU 2-x		
CT-scan	x	x	x	x		
Tumor specimen	x					
Physical examination	x	x	x	x		
Routine blood tests	x	x	x	x		
CA 19 − 9, AFP	x	x		x		
Blood samples investigation	x	x	x	x		
Quality of life survey	x	x	x	x		
Body weight	x	x	x	x		
Performance status	x	x	x	x		

Time points for acquisition of data from patients with HCC undergoing palliative locoregional treatment, primarily through transarterial chemoembolization (TACE). Inclusion and initial assessment are carried out at the surgical department, with repeated assessment after procedure and at follow-up, initially after 4–6 weeks. Ensuing evaluations for the study will lie in conjunction with clinical follow up, either every three months, or denser, depending on situation, for as long as additional TACE is considered

**Table 5:** HCC palliative advanced

Investigation	Pretreat	C 2–17	FU 3 mo	FU 6 mo	FU 9 mo	FU 12 mo	
CT-scan	x		x	x	x	x	
Tumor specimen	x						
Physical examination	x		x	x	x	x	
Routine blood tests	x	x					
CA 19 − 9, AFP	x		x	x	x	x	
Blood samples investigation	x	x					
Quality of life survey	x		x	x	x	x	
Body weight	x		x	x	x	x	
Performance status	x		x	x	x	x	

Time points for acquisition of data from patients with HCC undergoing palliative treatment. Inclusion and baseline assessment is carried out at the oncology department before start of treatment. Before each treatment cycle routine blood tests and additional investigational blood samples are gathered. CT-scans and clinical follow-up occur usually every three months during treatment. Last follow-up is after 12 months, or with cessation of treatment

### Research autopsies

All autopsies are registered as regular autopsies in the clinical pathology department and carried out as soon as possible after death, usually within 24 h, by a trained pathologist. Depending on the location and extent of the primary tumor and/or metastases, complete or parts of the affected organs will be removed and put in formalin prior to dissection. Tissue samples for formalin fixation and paraffin embedding may also be obtained during the autopsy. From lesions of sufficient size, tissue specimens will also be preserved by snap freezing.

## Single patient tissue chips

An individual tissue microarray (TMA), a so called “Single Patient Tissue Chip” (SPTC), will be constructed for patients with resected tumors and autopsied cases, as earlier described [32, 33] to enable comprehensive in situ mRNA and protein detection.

The SPTC contains several tissue cores (1 mm diameter) from all archival paraffin blocks from the primary tumor and any present lymph node metastases of sufficient size from resected cases. Normal tissue will be sampled from the resection margins of the primary tumor specimen. From autopsied patients, multiple samples will be obtained from the primary tumor in non-resected cases, from local recurrences in resected cases, and from all distant metastases of sufficient size. Alongside the construction of SPTCs, additional tissue cores will be taken from the immediately adjacent area of each TMA core for DNA and RNA extraction and sequencing.

### Genomic profiling

Genomic profiling of tissue will be carried out by targeted deep sequencing (TDS), using a broad panel covering > 500 cancer-related genes, for the detection of single nucleotide variants (SNVs), insertions and deletions (INDELs), copy number alterations (CNAs) and gene rearrangements. For quantification of ctDNA and monitoring of the temporal clonal dynamics, circulating cell free DNA will be extracted from plasma samples from all time points. Building on the genomic profiles in the tumor tissue, tailored panels will be applied for ctDNA profiling in plasma and for optimal detection of minimal residual disease. Broader panels and additional methodologies will be applied when relevant. The tumor mutational load will be calculated in both tissue and plasma samples.

### Circulating immune cells and other biomarkers

For assessment of circulating immune cells, freezing of buffy coat from the blood samples will be performed according to standard procedures. Flow cytometry will be carried out on all samples with multiple markers for T cell subsets, B cells, NK cell subsets, myeloid cell subsets and immune exhaustion. Building on results from the flow cytometry analyses, further studies will be carried out on selected cases, such as single cell RNA sequencing for in-depth characterization of particular circulating immune cell subsets of interest. Serum or plasma samples will be used to measure levels of immune-associated proteins and other relevant protein biomarkers using appropriate technologies.

### Quality control and assurance

The study will be conducted in accordance with the GCP guidelines for quality control and quality assurance. The web-based application REDCap will be used to manage all data generated, including an electronic case report form (eCRF).

Before start of treatment, the patients will receive information about the study by a physician and give their written informed consent.

### Data analysis

Overall and disease-free survival and time to progression in relation to different biomarkers and/or signatures thereof will be analyzed by the Kaplan-Meier method and the log-rank test. Hazard ratios for progression, relapse and death will be calculated with univariable and multivariable Cox regression models. Targeted and whole-exome sequencing will be followed by clonal deconvolution and the construction of cancer cell phylogenetic trees using e.g. the DEVOLUTION algorithm as previously described [34]. Individual somatic mutations and phylogenetic data will be correlated to ctDNA mutational profiles, clinical data, and other relevant biomarkers. RECIST criteria for ctDNA [35] response evaluation will be considered.

### Sample size considerations

Since the study is exploratory and there are no predefined hypotheses, no power calculations have been made. We estimate that the planned number of 150 study subjects and the extensive amount of samples obtained will be sufficient to uncover potential correlations or patterns that will generate new research questions and hypotheses to be tested in future studies.

## Discussion

Primary malignancies of the liver and biliary tract are aggressive and highly lethal forms of cancer, often with concomitant liver disease and other comorbidities that offer great challenge for both health care providers and society. The emergence of effective anti-viral therapies, for Hepatitis C in particular, and lifestyle changes, has altered the course, but increasing rates of metabolic dysfunction and ensuing liver disease leads in opposite direction. The treatment landscape, especially for HCC, has evolved markedly in the past five years, but a great number of patients do not benefit from available treatments. For CCC patients, the addition of check point inhibitors to chemotherapy offers only a modest improvement in overall survival, and most patients have no benefit. Targeted therapy is only applicable for a minority of patients. With our study, we wish to add pieces to the puzzle, by further elucidating how the molecular dynamics of individual tumors align with trajectories of treatment response, and patient outcome.

In HCC, several studies have been performed regarding cell free DNA (cfDNA) and ctDNA. Levels of cfDNA and ctDNA have been shown to correlate with tumor burden and to predict early recurrence after surgery [36–38] and response to Sorafenib in the palliative setting [39]. Presence of human telomerase reverse transcriptase (hTERT) gene promoter mutations is common in HCC and has been associated with worse outcome [40, 41]. Matsumae et al. evaluated pre-treatment cfDNA levels and ctDNA profile of 25 genes in patients about to undergo treatment with atezolizumab and bevacizumab and correlated them with outcome [42]. There was a marked difference in response based on cfDNA levels, with ORR 45.2% in the cfDNA low group, compared to 22.5% in the cfDNA high group. There was however no significant difference in PFS or OS with or without detectable ctDNA. The presence of TERT mutation indicated a worse prognosis, but no specific gene altering predicted therapeutic response [42]. The available data is mainly from trials with Asian patients. Mohamed et al. investigated baseline ctDNA mutations with correlation to OS and PFS in 44 patients treated with Nivolumab in Texas, USA. Mutations involving PIK3CA, BRCA1 and CCND1 were associated with shorter OS and mutations involving KIT and PIK3CA were associated with shorter PFS. Mutations in CTNNB1 were associated with longer PFS [43].

Studies evaluating cfDNA and ctDNA in CCC are less abundant and mainly restricted to those with intrahepatic localization. cfDNA levels and ctDNA alterations have been shown to provide information about prognosis and tumor burden [44–46]. Several studies have demonstrated genetic dynamics evaluated with ctDNA in patients receiving targeted therapy with FGFR2-fusion inhibitors and IDH-inhibitors, where acquired mutations and mechanisms of resistance were observed [47–51].

This prospective observational study will generate a unique biobank and thus enable research adding much needed knowledge on the molecular heterogeneity, also in a spatial and temporal context, of these many times hard-to-manage cancers. Since the majority of patients do not undergo surgery, studies of post-mortem collected tumor tissue will add substantial value, not least regarding the possibility to identify novel treatment targets.

We anticipate that the acquired information will be essential for developing clinically applicable models to predict the evolvability of the tumors, and to enable more precision in the selection of individualized treatment strategies, guided by liquid biopsies and appropriate biomarkers.

## Data Availability

No datasets were generated or analysed during the current study.

## Declarations

### Trial status

The study will be initiated in May 2025.

## Abbreviations

HCC: Hepatocellular carcinoma
CCC: Cholangiocellular carcinoma/cholangiocarcinoma
TKI: Tyrosine kinase inhibitors
ctDNA: Circulating tumour DNA
eCRF: Electronic case report form
FFPE: Formalin-fixed paraffin embedded
SPTC: Single Patient Tissue Chip
TDS: Targeted deep sequencing
TMA: Tissue microarray

## References

[1] Rumgay H, et al. Global, regional and National burden of primary liver cancer by subtype. Eur J Cancer. 2022;161:108–18. 34942552 10.1016/j.ejca.2021.11.023

[2] Massarweh NN, El-Serag HB. Epidemiology of hepatocellular carcinoma and intrahepatic cholangiocarcinoma. Cancer Control. 2017;24(3):1073274817729245. 28975830 10.1177/1073274817729245 PMC5937247

[3] Levercellscancer. *Nationellt vårdprogram*. 2022, Regionalt Cancercentrum i samverkan.

[4] Shin SW, et al. Liver resection versus local ablation therapies for hepatocellular carcinoma within the Milan criteria: A systematic review and Meta-analysis. Ann Surg. 2021;273(4):656–66. 33074898 10.1097/SLA.0000000000004350

[5] Finn RS, et al. Atezolizumab plus bevacizumab in unresectable hepatocellular carcinoma. N Engl J Med. 2020;382(20):1894–905. 32402160 10.1056/NEJMoa1915745

[6] Abou-Alfa GK, et al. Tremelimumab plus durvalumab in unresectable hepatocellular carcinoma. NEJM Evid. 2022;1(8):EVIDoa2100070. 38319892 10.1056/EVIDoa2100070

[7] Galle PR, et al. Nivolumab (NIVO) plus ipilimumab (IPI) vs lenvatinib (LEN) or Sorafenib (SOR) as first-line treatment for unresectable hepatocellular carcinoma (uHCC): first results from checkmate 9DW. J Clin Oncol. 2024;42(17suppl):LBA4008–4008.

[8] Chidambaranathan-Reghupaty S, Fisher PB, Sarkar D. Hepatocellular carcinoma (HCC): epidemiology, etiology and molecular classification. Adv Cancer Res. 2021;149:1–61. 33579421 10.1016/bs.acr.2020.10.001 PMC8796122

[9] Llovet JM, et al. Sorafenib in advanced hepatocellular carcinoma. N Engl J Med. 2008;359(4):378–90. 18650514 10.1056/NEJMoa0708857

[10] Clements O, et al. Risk factors for intrahepatic and extrahepatic cholangiocarcinoma: A systematic review and meta-analysis. J Hepatol. 2020;72(1):95–103. 31536748 10.1016/j.jhep.2019.09.007

[11] Petrick JL, et al. Risk factors for intrahepatic and extrahepatic cholangiocarcinoma in the united states: A population-based study in SEER-Medicare. PLoS ONE. 2017;12(10):e0186643. 29049401 10.1371/journal.pone.0186643 PMC5648218

[12] Shin HR, et al. Epidemiology of cholangiocarcinoma: an update focusing on risk factors. Cancer Sci. 2010;101(3):579–85. 20085587 10.1111/j.1349-7006.2009.01458.x PMC11158235

[13] Khan SA, Tavolari S, Brandi G. Cholangiocarcinoma: epidemiology and risk factors. Liver Int. 2019;39(Suppl 1):19–31. 30851228 10.1111/liv.14095

[14] Le VH, et al. Outcomes of neoadjuvant therapy for cholangiocarcinoma: A review of existing evidence assessing treatment response and R0 resection rate. J Surg Oncol. 2021;123(1):164–71. 32974932 10.1002/jso.26230

[15] Jeong H, et al. Adjuvant gemcitabine plus cisplatin versus capecitabine in node-positive extrahepatic cholangiocarcinoma: the STAMP randomized trial. Hepatology. 2023;77(5):1540–9. 37070950 10.1097/HEP.0000000000000046

[16] Valle J, et al. Cisplatin plus gemcitabine versus gemcitabine for biliary tract cancer. N Engl J Med. 2010;362(14):1273–81. 20375404 10.1056/NEJMoa0908721

[17] Kim ST, et al. Capecitabine plus oxaliplatin versus gemcitabine plus oxaliplatin as first-line therapy for advanced biliary tract cancers: a multicenter, open-label, randomized, phase III, noninferiority trial. Ann Oncol. 2019;30(5):788–95. 30785198 10.1093/annonc/mdz058

[18] Kelley RK, et al. Pembrolizumab in combination with gemcitabine and cisplatin compared with gemcitabine and cisplatin alone for patients with advanced biliary tract cancer (KEYNOTE-966): a randomised, double-blind, placebo-controlled, phase 3 trial. Lancet. 2023;401(10391):1853–65. 37075781 10.1016/S0140-6736(23)00727-4

[19] Oh DY, et al. Durvalumab plus gemcitabine and cisplatin in advanced biliary tract Cancer. NEJM Evid. 2022;1(8):EVIDoa2200015. 38319896 10.1056/EVIDoa2200015

[20] Abou-Alfa GK, et al. Pemigatinib for previously treated, locally advanced or metastatic cholangiocarcinoma: a multicentre, open-label, phase 2 study. Lancet Oncol. 2020;21(5):671–84. 32203698 10.1016/S1470-2045(20)30109-1 PMC8461541

[21] Zhu AX, et al. Final overall survival efficacy results of Ivosidenib for patients with advanced cholangiocarcinoma with IDH1 mutation: the phase 3 randomized clinical ClarIDHy trial. JAMA Oncol. 2021;7(11):1669–77. 34554208 10.1001/jamaoncol.2021.3836 PMC8461552

[22] Marabelle A, et al. Efficacy of pembrolizumab in patients with noncolorectal high microsatellite instability/mismatch Repair-Deficient cancer: results from the phase II KEYNOTE-158 study. J Clin Oncol. 2020;38(1):1–10. 31682550 10.1200/JCO.19.02105 PMC8184060

[23] Marabelle A, et al. Association of tumour mutational burden with outcomes in patients with advanced solid tumours treated with pembrolizumab: prospective biomarker analysis of the multicohort, open-label, phase 2 KEYNOTE-158 study. Lancet Oncol. 2020;21(10):1353–65. 32919526 10.1016/S1470-2045(20)30445-9

[24] Marcus L, et al. FDA approval summary: pembrolizumab for the treatment of tumor mutational Burden-High solid tumors. Clin Cancer Res. 2021;27(17):4685–9. 34083238 10.1158/1078-0432.CCR-21-0327 PMC8416776

[25] Subbiah V, et al. Dabrafenib plus Trametinib in patients with BRAF(V600E)-mutated biliary tract cancer (ROAR): a phase 2, open-label, single-arm, multicentre basket trial. Lancet Oncol. 2020;21(9):1234–43. 32818466 10.1016/S1470-2045(20)30321-1

[26] Javle M, et al. Pertuzumab and trastuzumab for HER2-positive, metastatic biliary tract cancer (MyPathway): a multicentre, open-label, phase 2a, multiple basket study. Lancet Oncol. 2021;22(9):1290–300. 34339623 10.1016/S1470-2045(21)00336-3

[27] Nakamura Y, et al. Tucatinib and trastuzumab for previously treated human epidermal growth factor receptor 2-Positive metastatic biliary tract Cancer (SGNTUC-019): A phase II basket study. J Clin Oncol. 2023;41(36):5569–78. 37751561 10.1200/JCO.23.00606 PMC10730072

[28] Meric-Bernstam F, et al. Efficacy and safety of trastuzumab Deruxtecan in patients with HER2-Expressing solid tumors: primary results from the DESTINY-PanTumor02 phase II trial. J Clin Oncol. 2024;42(1):47–58. 37870536 10.1200/JCO.23.02005 PMC10730032

[29] Drilon A, et al. Efficacy of larotrectinib in TRK Fusion-Positive cancers in adults and children. N Engl J Med. 2018;378(8):731–9. 29466156 10.1056/NEJMoa1714448 PMC5857389

[30] Brunt E, et al. cHCC-CCA: consensus terminology for primary liver carcinomas with both hepatocytic and cholangiocytic differentation. Hepatology. 2018;68(1):113–26. 29360137 10.1002/hep.29789 PMC6340292

[31] Xue R, et al. Genomic and transcriptomic profiling of combined hepatocellular and intrahepatic cholangiocarcinoma reveals distinct molecular subtypes. Cancer Cell. 2019;35(6):932–e9478. 31130341 10.1016/j.ccell.2019.04.007 PMC8317046

[32] Hau SO, et al. Chemotherapy, host response and molecular dynamics in periampullary cancer: the CHAMP study. BMC Cancer. 2020;20(1):308. 32293352 10.1186/s12885-020-06807-3 PMC7161011

[33] Petersson A, et al. Branching Copy-Number evolution and parallel immune profiles across the regional tumor space of resected pancreatic Cancer. Mol Cancer Res. 2022;20(5):749–61. 35149544 10.1158/1541-7786.MCR-21-0986 PMC9381114

[34] Andersson N, et al. DEVOLUTION-A method for phylogenetic reconstruction of aneuploid cancers based on multiregional genotyping data. Commun Biol. 2021;4(1):1103. 34545199 10.1038/s42003-021-02637-6 PMC8452746

[35] Jakobsen AKM, Spindler KG. ctDNA-Response evaluation criteria in solid tumors - a new measure in medical oncology. Eur J Cancer. 2023;180:180–3. 36610263 10.1016/j.ejca.2022.11.039

[36] Tokuhisa Y, et al. Circulating cell-free DNA as a predictive marker for distant metastasis of hepatitis C virus-related hepatocellular carcinoma. Br J Cancer. 2007;97(10):1399–403. 17940509 10.1038/sj.bjc.6604034 PMC2360234

[37] Cai Z, et al. Comprehensive liquid profiling of Circulating tumor DNA and protein biomarkers in Long-Term Follow-Up patients with hepatocellular carcinoma. Clin Cancer Res. 2019;25(17):5284–94. 31217202 10.1158/1078-0432.CCR-18-3477

[38] Zhu GQ, et al. Serial Circulating tumor DNA to predict early recurrence in patients with hepatocellular carcinoma: a prospective study. Mol Oncol. 2022;16(2):549–61. 34543520 10.1002/1878-0261.13105 PMC8763657

[39] Oh CR, et al. Genome-wide copy number alteration and VEGFA amplification of Circulating cell-free DNA as a biomarker in advanced hepatocellular carcinoma patients treated with Sorafenib. BMC Cancer. 2019;19(1):292. 30935424 10.1186/s12885-019-5483-x PMC6444867

[40] Ako S, et al. Human telomerase reverse transcriptase gene promoter mutation in serum of patients with hepatocellular carcinoma. Oncology. 2020;98(5):311–7. 32135540 10.1159/000506135

[41] Hirai M, et al. Prediction of the prognosis of advanced hepatocellular carcinoma by TERT promoter mutations in Circulating tumor DNA. J Gastroenterol Hepatol. 2021;36(4):1118–25. 32830343 10.1111/jgh.15227

[42] Matsumae T et al. Circulating Cell-Free DNA profiling predicts the therapeutic outcome in advanced hepatocellular carcinoma patients treated with combination immunotherapy. Cancers (Basel), 2022. 14(14). 10.3390/cancers14143367 PMC9320668 35884434

[43] Mohamed YI, et al. Circulating tumor DNA (ctDNA) as a biomarker of response to therapy in advanced hepatocellular carcinoma treated with nivolumab. Cancer Biomark. 2024;41(1):83–91. 39269823 10.3233/CBM-230431 PMC11491993

[44] Uson Junior PLS, et al. Cell-Free tumor DNA dominant clone allele frequency is associated with poor outcomes in advanced biliary cancers treated with Platinum-Based chemotherapy. Volume 6. JCO Precis Oncol; 2022. p. e2100274. 1. 10.1200/PO.21.00274 PMC9200394 35666960

[45] Winter H et al. Identification of Circulating genomic and metabolic biomarkers in intrahepatic cholangiocarcinoma. Cancers (Basel), 2019. 11(12). 10.3390/cancers11121895 PMC6966597 31795195

[46] Wintachai P et al. Diagnostic and prognostic value of Circulating Cell-Free DNA for cholangiocarcinoma. Diagnostics (Basel), 2021. 11(6). 10.3390/diagnostics11060999 PMC8228499 34070951

[47] Varghese AM, et al. Noninvasive detection of polyclonal acquired resistance to FGFR Inhibition in patients with cholangiocarcinoma harboring FGFR2 alterations. JCO Precis Oncol; 2021. p. 5. 10.1200/PO.20.00178 PMC8232836 34250419

[48] Goyal L, et al. TAS-120 overcomes resistance to ATP-Competitive FGFR inhibitors in patients with FGFR2 Fusion-Positive intrahepatic cholangiocarcinoma. Cancer Discov. 2019;9(8):1064–79. 31109923 10.1158/2159-8290.CD-19-0182 PMC6677584

[49] Cleary JM, et al. Secondary IDH1 resistance mutations and oncogenic IDH2 mutations cause acquired resistance to Ivosidenib in cholangiocarcinoma. NPJ Precis Oncol. 2022;6(1):61. 36056177 10.1038/s41698-022-00304-5 PMC9440204

[50] Lapin M, et al. Monitoring of dynamic changes and clonal evolution in Circulating tumor DNA from patients with IDH-Mutated cholangiocarcinoma treated with isocitrate dehydrogenase inhibitors. Volume 6. JCO Precis Oncol; 2022. p. e2100197. 10.1200/PO.21.00197 PMC8865526 35171660

[51] Wu Q, et al. Landscape of clinical resistance mechanisms to FGFR inhibitors in FGFR2-Altered cholangiocarcinoma. Clin Cancer Res. 2024;30(1):198–208. 37843855 10.1158/1078-0432.CCR-23-1317 PMC10767308

